# Effect of Hemodialysis on Anterior Chamber Angle Measured by Anterior Segment Optical Coherence Tomography

**DOI:** 10.1155/2019/2406547

**Published:** 2019-08-14

**Authors:** Yong Un Shin, Ji Hong Kim, Heeyoon Cho, Dae Sung Kim, Joo-Hark Yi, Sang-Woong Han, Mincheol Seong

**Affiliations:** ^1^Department of Ophthalmology, Hanyang University College of Medicine, Seoul, Republic of Korea; ^2^Division of Nephrology, Department of Internal Medicine, Hanyang University College of Medicine, Seoul, Republic of Korea

## Abstract

**Purpose:**

To investigate the effect of hemodialysis (HD) on the anterior chamber angle by anterior segment optical coherence tomography (ASOCT) and other ophthalmologic parameters in patients with end-stage kidney disease (ESKD).

**Methods:**

A prospective observational study was performed on 20 patients who underwent HD for ESKD. Anterior chamber angle images were obtained by 16 mm line scan of ASOCT. The angle opening distance (AOD) and the trabecular-iris space area (TISA) were determined using the ImageJ program. Additional 12 mm horizontal and 9 mm vertical wide-field scans centered on the posterior pole were performed for the measurement of peripapillary retinal nerve fiber layer (pRNFL) thickness and macular ganglion cell-inner plexiform layer (mGCIPL) thickness. Changes in intraocular pressure (IOP), AOD, TISA, pRNFL thickness, and mGCIPL thickness before and after HD were statistically analyzed.

**Results:**

The IOP decreased significantly from 17.5 ± 3.4 before HD to 16.2 ± 2.3 after HD (*P*=0.017). There was a statistically significant decrease in AOD 750 and TISA 750 (*P*=0.005 and *P*=0.007, respectively). AOD 500 and TISA 500 also decreased, which was almost statistically significant (*P*=0.061 and *P*=0.081, respectively). Mean pRNFL thickness and mGCIPL thickness did not show significant change after HD.

**Conclusion:**

We observed a significant decrease in IOP and anterior chamber angle measurements after HD. Our study suggests that HD can influence the anterior segment structure of eyes.

## 1. Introduction

Chronic kidney disease is a condition that occurs due to a permanent reduction in glomerular filtration rate and irreversible renal dysfunction. End-stage kidney disease (ESKD) is defined as a glomerular filtration rate less than 15 ml/min/1.73 m^2^ and requires renal replacement therapy, such as dialysis or renal transplantation. Hemodialysis (HD) is a common renal replacement therapy, which removes excessive fluid and uremic substances, thereby balancing the volume composition of the body fluids.

Dynamic changes in body fluid composition due to HD result in systemic changes, such as decreases in body weight and systolic blood pressure [1, 2]. In addition to these systemic changes, HD has been reported to exhibit various effects on the eye, such as refractive changes, dry eye, conjunctival calcium deposits, band keratopathy, lenticular opacities, and changes in intraocular pressure (IOP) [3–6]. Recently, there have been several reports on changes in retinal and choroidal thickness before and after HD using optical coherence tomography [7–10].

Hemodynamic changes due to HD may affect the composition of aqueous humor, which may influence the structure of the anterior chamber angle. However, the effect of HD on the anterior chamber angle has not been reported. In addition, few reports have examined the changes in peripapillary retinal nerve fiber layer (pRNFL) and macular ganglion cell-inner plexiform layer (mGCIPL) after HD.

In this study, we investigated the effect of HD on the anterior chamber angle in patients with ESKD undergoing HD through anterior segment optical coherence tomography (ASOCT). Furthermore, we also investigated the changes in pRNFL and mGCIPL thickness after HD by swept-source optical coherence tomography.

## 2. Methods

### 2.1. Patients

Our study protocol complied with the Declaration of Helsinki and was approved by the Institutional Review Board (IRB) of Hanyang University Guri Hospital. A prospective observational study of ESKD patients undergoing HD at Hanyang University Guri Hospital was performed (IRB no. 2016-05-005). We recruited patients who were regularly dialyzed in our dialysis room to participate in the study. All patients underwent baseline measurement of body weight and blood pressure before and after HD. After detailed explanation of the study, informed consent was obtained from all participants.

Exclusion criteria were as follows: (1) previous history of intraocular surgery or glaucoma diagnosis, (2) baseline IOP greater than 22 mmHg, (3) presence of glaucomatous optic disc changes including excavation, thinning, or notching of the neuroretinal rim, (4) closed or occludable angle in gonioscopic examination, (5) axial length greater than 26 mm, (6) combined anterior segment disease such as corneal opacity, and (7) combined macular and retinal diseases such as central serous chorioretinopathy and age-related macular degeneration, except diabetic retinopathy.

### 2.2. Ocular Examinations

The right eye was selected for ophthalmic examination to shorten the interval between HD and examinations. All ophthalmologic examinations were performed in the dialysis room to exclude spurious effects. To do so, all ophthalmic devices were placed at the entrance of the dialysis room. In particular, the OCT was installed in a separately shaded area, minimizing changes in pupil size due to illumination. At the beginning of HD, all patients underwent comprehensive ophthalmic examinations including best-corrected visual acuities, slit-lamp examinations, gonioscopy, IOP, anterior chamber depth (ACD), axial length (AL), and indirect ophthalmoscopy. IOP was measured three times in succession using a TonoPen® (Reichert Inc., Depew, NY, USA), and the mean value of measurements was used for the study. ACD and AL were measured using an IOL master® (Carl Zeiss, Jena, Germany).

OCT was performed by swept-source optical coherence tomography (DRI-OCT Triton®, Topcon Inc., Tokyo, Japan). The device can be extended to include anterior imaging. To obtain images of the same area before and after HD, the patient's nasal and temporal limbus were marked with a marking pen. After positioning the anterior segment attachment on the OCT device, anterior chamber angle images were obtained by performing a 16 mm line scan to include the two points marked. In addition, posterior pole imaging was performed to obtain pRNFL thickness and mGCIPL thickness. Using a 1,050 nm wavelength light source, the device provides wide-field three-dimensional macular volume scanning protocols, covering a 12 mm × 9 mm area on the posterior pole, including the macular and peripapillary areas. After HD, systemic parameters such as body weight and blood pressure and ophthalmic parameters such as IOP, ACD, AL, and OCT measurements were measured again. The interval between ophthalmologic examinations and HD was less than 10 minutes.

### 2.3. Analysis of OCT Measurements

**Figure 1:**
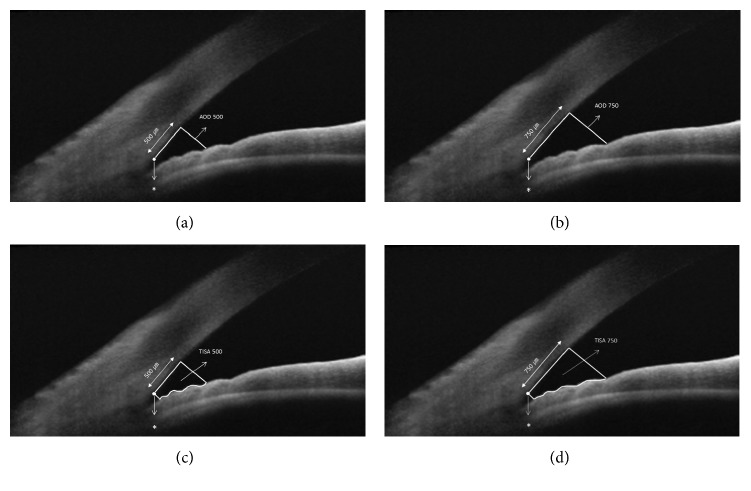
Measurement of quantitative angle parameters using ASOCT. (a, b) Angle opening distance (AOD). (c, d) Trabecular-iris space area (TISA). AOD 500 and 750 (angel opening distance): a distance between a point of the cornea which is 500 and 750 *μ*m away from the scleral spur and the opposite point of the iris. TISA 500 and 750 (trabecular-iris space area): an area covering 500 and 750 *μ*m located in the area bounded by the cornea and the iris.

The images obtained through ASOCT were exported and saved as Tiff files via OCT viewer software. We loaded the exported image into the ImageJ program (software version 1.46; National Institutes of Health, Bethesda, MD, USA). Postprocessing of images was performed to clearly identify the structure of the anterior chamber angle. For analysis of ASOCT measurements, the following parameters were calculated using the ImageJ program: (1) Angle opening distance (AOD 500 or AOD 750), the distance between a point on the cornea 500 or 750 *μ*m from the scleral spur and the opposite point of the iris, and (2) trabecular-iris space area (TISA 500 or TISA 750), an area covering 500 or 750 *μ*m located in the area bounded by the cornea and the iris [11] (Figure 1). All images obtained through ASCOT were evaluated for their quality according to the visibility of the scleral spur. In some subjects, scleral spur was not observed due to posterior shadowing, with strong signal formation at the corneoscleral junction. In other cases, despite the excellent visibility, a typical inward protrusion of the scleral spur was not observed on the inner surface of the sclera. Likewise, all images that could not identify the location of the scleral spur were excluded from the study.

The pRNFL thickness measurements in the 12 subfields (superotemporal, superior, superonasal, nasosuperior, nasal, nasoinferior, inferonasal, inferior, inferotemporal, temporoinferior, temporal, and temporosuperior) were performed using RNFL thickness map analysis protocols. The mGCIPL thickness was automatically measured as the distance between the inner border of the ganglion cell layer and the outer border of the inner plexiform layer. After checking for segmentation error, the mGCIPL thickness map on the macula six sector grids was then obtained using the ganglion cell analysis algorithm, which provided the average of six sectors (superotemporal, superior, superonasal, inferonasal, inferior, and inferotemporal) of the elliptical annulus. All scans of the posterior segment were obtained with a minimum signal strength index of 50 and above.

### 2.4. Statistical Analysis

We performed all statistical analyses using SPSS for Windows version 18.0 (SPSS, Inc., Chicago, IL, USA). The Wilcoxon signed-rank test was used to determine changes in systemic parameters, such as body weight and blood pressure, and ophthalmic parameters, such as IOP, ACD, AL, and OCT measurements before and after HD. Continuous data were presented as the mean ± standard deviation. *P* values less than 0.05 were considered significant.

## 3. Results

### 3.1. Demographic and Clinical Characteristics

A total of 20 ESKD patients undergoing HD participated in the study.  Table 1 shows the demographic data and clinical characteristics of the included patients. The mean age of the patients was 53.6 ± 10.9 years, and 10 men and 10 women were included. Hypertension was found in 14 patients (70.0%), and diabetes mellitus was found in eight patients (40.0%). The most common causes of HD were hypertensive (30.0%) and diabetic nephropathy (30.0%). Mean body weight was 63.8 ± 11.0 kg before HD and 61.0 ± 11.2 kg after HD. The decrease in body weight was statistically significant (*P* < 0.001, Wilcoxon signed-rank test). The mean systolic and diastolic blood pressures before HD were 160.2 ± 18.8 mmHg and 77.2 ± 11.3 mmHg, respectively. The systolic blood pressure after HD decreased significantly to 149.5 ± 20.4 mmHg (*P*=0.040). However, diastolic blood pressure did not show a statistically significant change (*P*=0.111).

### 3.2. Changes in Anterior Chamber Angle after Hemodialysis

Table 2 shows changes in IOP, ACD, AL, and anterior chamber angle parameters before and after HD. IOP decreased significantly from 17.5 ± 3.4 before HD to 16.2 ± 2.3 after HD (*P*=0.017). ACD and AL showed little change after HD. Significant changes in parameters of the anterior chamber angle measured with ASOCT were observed. AOD 750 showed a statistically significant decrease from 0.647 ± 0.253 mm before HD to 0.581 ± 0.249 mm after HD (*P*=0.005). Similarly, TISA 750 also decreased significantly from 0.326 ± 0.123 mm^2^ to 0.299 ± 0.122 mm^2^ after HD (*P*=0.007). AOD 500 and TISA 500 also decreased after HD, but the difference was not statistically significant (*P*=0.061 and *P*=0.081, respectively).

### 3.3. Peripapillary RNFL Thickness and Macular GCIPL Thickness before and after Hemodialysis

Table 3 shows changes in pRNFL thickness and mGCIPL thickness after HD. Mean pRNFL thickness did not change significantly from 98.1 ± 15.2 *μ*m before HD to 98.8 ± 15.3 *μ*m after HD (*P*=0.236). The average mGCIPL thickness was measured as 63.6 ± 6.9 *μ*m before HD and 64.0 ± 8.0 *μ*m after HD, which was also not statistically significant (*P*=0.499).

## 4. Discussion

Our results showed a significant decrease in IOP and anterior chamber angle measurements after HD. There was significant decrease in AOD 750 and TISA 750, and AOD 500 and TISA 500 showed a tendency to decrease after HD. Mean pRNFL thickness and mGCIPL thickness did not show significant change after HD.

Because hemodynamic changes due to HD have been expected to occur in the retina and choroid, which are relatively blood-rich tissues in the eye, most OCT studies have focused on retinal thickness and choroidal thickness [7–10]. In addition, most ophthalmologic studies related to HD examined the change in IOP [6,12–18]. The changes in IOP associated with HD have shown very diverse results, and various hypotheses have been reported to illustrate this variability. The structure of the anterior chamber angle, the site of aqueous humor release, may be an important factor to explain the change in IOP associated with HD. However, the analysis of anterior chamber angle images through ASOCT in HD patients has not yet been done. Although not HD, there has been a recent study of ASOCT in patients undergoing nocturnal intermittent peritoneal dialysis [19]. As a result, IOP decreased significantly, but no significant changes were observed in ASOCT parameters, such as AOD 500, AOD 750, TISA 500, and TISA 750, after nocturnal intermittent peritoneal dialysis. This was not consistent with our findings in HD patients.

In order to account for changes in the anterior chamber angle, consideration of IOP and aqueous humor is essential. Early studies have reported increased IOP due to increased production of aqueous humor [12, 20]. The reason for the elevation of IOP after HD was explained by the rapid decrease of plasma osmotic pressure or the increase of relative urea concentration in the aqueous humor, resulting in migration of extracellular fluid from the blood into the anterior chamber. Tawara and associates reported a change in IOP after HD between groups with and without compromise in aqueous outflow facility and observed a significant increase in IOP in the outflow obstruction group [21]. In the same way as the abovementioned studies, increased aqueous humor in the anterior chamber can increase IOP, which can be prevented by enhancing aqueous humor outflow. If there is no obstruction in aqueous outflow, IOP elevation will not occur due to this mechanism.

On the other hand, several studies recently reported a decrease in IOP after HD [7, 10, 16]. Tokuyama and associates evaluated the relationship between IOP and plasma colloid osmotic pressure [16]. In their study, IOP, plasma osmolarity, and body weight decreased significantly after HD, but plasma colloid osmotic pressure showed a significant increase after HD. Significant correlations were found between changes in IOP and that of plasma colloid osmotic pressure, not between changes in IOP and that of plasma osmolarity.

Our results showed significant decrease in IOP and body weight, which is similar to that of Tokuyama and associates. Unfortunately, we were unable to perform blood sampling before and after HD; therefore, plasma osmolality and plasma colloid osmotic pressure values could not be obtained. However, a decrease in body weight and hemodynamic changes, such as a decrease in systolic blood pressure, indirectly indicate a decrease in body fluid volume, which may lead to an increase in plasma colloid osmotic pressure. In addition, IOP elevation due to an abrupt decrease in plasma osmolarity is not expected to occur due to the recent development of dialysis technology. This is supported by the recent decline in IOP observed in most HD studies.

Prior to the development of OCT, attempts were made to observe changes in the anterior chamber angle of HD patients through gonioscopy. Jaeger and associates reported a narrow angle in patients with acute elevation of IOP after HD [22]. De Marchi and associates reported that IOP was elevated in patients with a narrow angle, whereas IOP did not change or decrease in patients with a normal angle [23]. In our study using ASOCT, the anterior chamber angle tended to be narrowed and the IOP decreased, which are different from previous studies. This discrepancy can be explained by the difference of interpretation according to cause and effect. As in other studies, narrow chamber angles may have the effect of increasing IOP by suppressing aqueous outflow, but our study suggests that a narrow angle was observed as a result of a decrease in aqueous humor, which leads to a decrease in IOP. As the plasma volume in the body decreases due to the exit of water from the plasma to the dialysate during HD, the concentration of plasma protein is relatively increased. As a result, the plasma colloid osmotic pressure is increased and water moves from the aqueous humor to the plasma, which leads to a decrease aqueous humor.

A decrease in aqueous humor can be expressed as a decrease in ACD, as well as a decrease in IOP and narrowing of the chamber angle. However, our results show that the ACD remained almost unchanged. This may suggest that changes in the anterior chamber angle, where the production and absorption of aqueous humor occur, are more vulnerable to changes in aqueous humor than to the central portion of the aqueous chamber. On the other hand, there may be discrepancies in the results of angle and center of anterior chamber due to differences in test methods. Since our ASOCT protocol cannot measure the ACD, we used the IOLmaster for ACD measurements. If it is possible to obtain an image for the entire anterior chamber, this error may be reduced.

In the present study, pRNFL thickness and mGCIPL thickness did not change after HD. These results were consistent with a previous study [24]. Demir and associates reported a decrease in RNFL thickness in chronic kidney disease patients receiving HD compared to the control group [25]. It is possible that RNFL thickness may be reduced in association with the death of retinal ganglion cells due to uremic optic neuropathy observed in chronic kidney disease and ischemic optic neuropathy caused by hypotension after HD [26, 27]. In this study, only the acute effects of HD were examined, which lead to no significant changes in pRNFL thickness or mGCIPL thickness.

Our study has some limitations. Firstly, as mentioned above, we could not measure changes in plasma osmolarity and plasma colloid osmotic pressure because we did not perform blood sampling before and after HD. Therefore, it was only possible to estimate these indirectly through changes in body weight and blood pressure. Secondly, IOP could not be measured with the Goldmann applanation tonometer, which is the gold standard method of IOP measurement. However, since the IOP measurements using TonoPen® were made in the bedside just before and immediately after HD, the interval between IOP measurements and HD was minimized, which made it possible to demonstrate the direct effect of HD on IOP. Furthermore, TonoPen has been shown to be as accurate as the Goldmann applanation tonometer when measuring IOP in adults with normal values [28]. Thirdly, because the study sample size is small and enrolled patients had various underlying diseases, this result may be difficult to apply to all patients undergoing HD. Therefore, future studies should include a large number of patients in order to demonstrate the relationships between changes in IOP, anterior chamber angle, plasma osmolarity, and plasma colloid osmotic pressure after HD.

In conclusion, we suggest that loss of body fluids after HD causes a decrease in body weight and a decrease in blood pressure; as a result, water is moved from the anterior chamber to the plasma via elevated plasma colloid osmotic pressure. This leads to a decrease in IOP and anatomical narrowing of the anterior chamber. Our results are novel because the structural changes in the anterior chamber angle in HD patients were first demonstrated by ASOCT with changes in IOP.

## Figures and Tables

**Table 1:** Demographic data and baseline clinical characteristics of patients.

Characteristics	Numbers
Age, years	53.6 ± 10.9
Sex, male : female	10 : 10
Hypertension	14 (70.0%)
Diabetes	8 (40.0%)
Cause of HD	
Hypertensive nephropathy	6 (30.0%)
Diabetic nephropathy	6 (30.0%)
IgA nephropathy	3 (15.0%)
Polycystic kidney disease	1 (5.0%)
Unknown	4 (20.0%)
Body weight before HD (kg)	63.8 ± 11.0
Body weight after HD (kg)	61.0 ± 11.2
*P* value	<0.001
Systolic blood pressure before HD (mmHg)	160.2 ± 18.8
Systolic blood pressure after HD (mmHg)	149. 5 ± 20.4
*P* value	0.040
Diastolic blood pressure before HD (mmHg)	77.2 ± 11.3
Diastolic blood pressure after HD (mmHg)	83.2 ± 10.7
*P* value	0.111

HD = hemodialysis.

**Table 2:** Comparison of anterior chamber angle parameters before and after hemodialysis.

Characteristics	Before HD	After HD	*P* value
IOP (mmHg)	17.5 ± 3.4	16.2 ± 2.3	0.017
ACD (mm)	3.3 ± 0.6	3.2 ± 0.5	0.367
AL (mm)	23.5 ± 1.3	23.6 ± 1.3	0.927
AOD 500 (mm)	0.459 ± 0.181	0.432 ± 0.187	0.061
AOD 750 (mm)	0.647 ± 0.253	0.581 ± 0.249	0.005
TISA 500 (mm^2^)	0.170 ± 0.065	0.162 ± 0.066	0.081
TISA 750 (mm^2^)	0.326 ± 0.123	0.299 ± 0.122	0.007

HD = hemodialysis; IOP = intraocular pressure; ACD = anterior chamber depth; AL = axial length; AOD = angle open distance; TISA = trabecular-iris space area.

**Table 3:** Comparison of peripapillary retinal nerve fiber layer thickness and macular ganglion cell-inner plexiform layer thickness before and after hemodialysis.

Characteristics	Before HD	After HD	*P* value
pRNFL thickness (*μ*m)			
Superotemporal	130.4 ± 33.7	132.6 ± 33.8	0.373
Superior	106.8 ± 23.4	108.2 ± 25.5	0.456
Superonasal	97.4 ± 18.4	98.8 ± 19.2	0.353
Nasosuperior	81.5 ± 18.5	82.6 ± 20.1	0.610
Nasal	68.0 ± 18.0	71.8 ± 15.2	0.858
Nasoinferior	70.7 ± 15.5	73.2 ± 17.4	0.288
Inferonasal	106.6 ± 24.4	105.8 ± 24.1	0.654
Inferior	138.3 ± 33.1	131.3 ± 41.5	0.094
Inferotemporal	132.6 ± 33.9	134.0 ± 35.7	0.401
Temporoinferior	77.6 ± 21.0	80.2 ± 22.8	0.241
Temporal	71.2 ± 15.7	72.1 ± 14.1	0.531
Temporosuperior	95.1 ± 24.8	99.3 ± 30.8	0.141
Average	98.1 ± 15.2	98.8 ± 15.3	0.236

mGCIPL thickness			
Superotemporal	63.4 ± 7.3	63.5 ± 9.4	0.902
Superior	63.3 ± 6.5	63.8 ± 8.8	0.520
Superonasal	67.9 ± 7.5	67.6 ± 9.6	0.832
Inferonasal	64.8 ± 9.2	65.4 ± 9.2	0.042
Inferior	58.8 ± 7.1	59.6 ± 6.9	0.019
Inferotemporal	63.3 ± 7.9	63.6 ± 8.7	0.501
Average	63.6 ± 6.9	64.0 ± 8.0	0.499

HD = hemodialysis; pRNFL = peripapillary retinal nerve fiber layer; mGCIPL = macular ganglion cell-inner plexiform layer.

## Data Availability

The data used to support the findings of this study are included within the article.
